# A clone-free, single molecule map of the domestic cow (*Bos taurus*) genome

**DOI:** 10.1186/s12864-015-1823-7

**Published:** 2015-08-28

**Authors:** Shiguo Zhou, Steve Goldstein, Michael Place, Michael Bechner, Diego Patino, Konstantinos Potamousis, Prabu Ravindran, Louise Pape, Gonzalo Rincon, Juan Hernandez-Ortiz, Juan F. Medrano, David C. Schwartz

**Affiliations:** Laboratory for Molecular and Computational Genomics, Department of Chemistry, Laboratory of Genetics, and the UW Biotechnology Center, University of Wisconsin-Madison, 425 Henry Mall, Madison, WI 53706 USA; Departamento de Materiales, Facultad de Minas, Universidad Nacional de Colombia, Sede Medellin, Calle 75 # 79A-51, Bloque M17, Medellin, Colombia, SA; Department of Animal Science, University of California-Davis, Davis, CA 95616 USA

## Abstract

**Background:**

The cattle (*Bos taurus*) genome was originally selected for sequencing due to its economic importance and unique biology as a model organism for understanding other ruminants, or mammals. Currently, there are two cattle genome sequence assemblies (UMD3.1 and Btau4.6) from groups using dissimilar assembly algorithms, which were complemented by genetic and physical map resources. However, past comparisons between these assemblies revealed substantial differences. Consequently, such discordances have engendered ambiguities when using reference sequence data, impacting genomic studies in cattle and motivating construction of a new optical map resource--BtOM1.0--to guide comparisons and improvements to the current sequence builds. Accordingly, our comprehensive comparisons of BtOM1.0 against the UMD3.1 and Btau4.6 sequence builds tabulate large-to-immediate scale discordances requiring mediation.

**Results:**

The optical map, BtOM1.0, spanning the *B. taurus* genome (Hereford breed, L1 Dominette 01449) was assembled from an optical map dataset consisting of 2,973,315 (439 X; raw dataset size before assembly) single molecule optical maps (Rmaps; 1 Rmap = 1 restriction mapped DNA molecule) generated by the Optical Mapping System. The BamHI map spans 2,575.30 Mb and comprises 78 optical contigs assembled by a combination of iterative (using the reference sequence: UMD3.1) and *de novo* assembly techniques. BtOM1.0 is a high-resolution physical map featuring an average restriction fragment size of 8.91 Kb. Comparisons of BtOM1.0 *vs.* UMD3.1, or Btau4.6, revealed that Btau4.6 presented far more discordances (7,463) *vs.* UMD3.1 (4,754). Overall, we found that Btau4.6 presented almost double the number of discordances than UMD3.1 across most of the 6 categories of sequence *vs.* map discrepancies, which are: COMPLEX (misassembly), DELs (extraneous sequences), INSs (missing sequences), ITs (Inverted/Translocated sequences), ECs (extra restriction cuts) and MCs (missing restriction cuts).

**Conclusion:**

Alignments of UMD3.1 and Btau4.6 to BtOM1.0 reveal discordances commensurate with previous reports, and affirm the NCBI’s current designation of UMD3.1 sequence assembly as the “reference assembly” and the Btau4.6 as the “alternate assembly.” The cattle genome optical map, BtOM1.0, when used as a comprehensive and largely independent guide, will greatly assist improvements to existing sequence builds, and later serve as an accurate physical scaffold for studies concerning the comparative genomics of cattle breeds.

**Electronic supplementary material:**

The online version of this article (doi:10.1186/s12864-015-1823-7) contains supplementary material, which is available to authorized users.

## Background

Cattle are the most common type of large domesticated animals and have consequently played an important role in recent history of humankind since their domestication 8,000 to 10,000 years ago [1]. Cattle have enhanced human civilizations through their varied uses as livestock for meat, milk, and draft power. Accordingly, there are ~1.3 billion cattle in the world today providing a significant source of nutrition and livelihood to the human population. Domestic cattle comprise more than 800 breeds and are grouped taxonomically into two species—*Bos taurus* (taurine) and *B. indicus* (indicine)—which were evolved from the ancestral species of *B. primigenius*. Given this large and venerable resource of cattle breeds, cattle research efforts have also greatly contributed to our knowledge of genetics, endocrine function, fertilization, growth, lactation and mammalian biology. As such, there are still many unsolved questions regarding cattle adaptation to diverse terrestrial environments since domestication that center on how cattle convert low-grade forage to energy-rich fat, milk and meat, and, more fundamentally, how genetic underpinnings define economically important traits. The cattle genome was originally selected for sequencing due to its unique biology and economic importance, virtues that are also strengthened by its role as a model organism for understanding other ruminants, or mammals.

The Bovine Genome Sequencing and Analysis Consortium published the first draft sequence for the *Bos taurus* genome in 2009--a sizable effort costing $53 million and involving nearly 300 investigators from 25 countries [2, 3]. The initial sequence assembly (Btau4.0) was constructed by the Baylor College of Medicine Human Genome Sequencing Center using ~7.1-fold Sanger sequencing coverage of the genome. Their genome assembly approach combined a BAC (Bacterial Artificial Chromosome) clone-by-clone approach with whole genome shotgun (WGS) reads, and yielded an N50 contig size of 48.7 Kb and a N50 scaffold size of 1.9 Mb (Btau4.0; 135,743 contigs; 13,388 scaffolds; total mass: 2.77 Gb). 89 % of these assembled contigs and scaffolds were anchored onto the 29 bovine autosomes and the X chromosome based on the integrated FPC physical map [4], which combined a series of complementary mapping resources: 290,797 fingerprinted BACs, the human-cattle comparative map, the genetic map, and the radiation hybrid (RH) map [2–10]. The Center for Bioinformatics and Computational Biology, University of Maryland, using a different strategy, constructed another bovine assembly in 2009 based on the same raw sequence and map data (UMD2; 44,433 contigs; total mass: 2.86 Gb; contig N50: 93.56 Kb). Their strategy leveraged paired-end BAC sequence information, mapping data and, most notably, syntenic relationships to the human genome that allowed 91 % of the UMD2 contigs to be anchored to bovine chromosomes, based on the integrated bovine genome map [4, 11]. Comparisons between these two assemblies revealed substantial differences that appear as assembly errors, genome segmental inversions, chromosomal placements, sequence gap numbers, and discrepancies of the sequence coverage across the bovine genome [11–13]. Two updated bovine genome sequence assemblies (Btau4.6 and UMD3.1) were released from these groups featuring additional BAC sequence data, corrected assembly errors and additional gap filling. Although comprehensive analyses of these recent releases have yet to be done, significant differences between these updated assemblies are generally expected to be encountered. Indeed, this article reports on notable disparities. Consequently, discrepancies between these assemblies engender ambiguities when using reference sequence data, which significantly impacts almost any type of genomic study in cattle.

The cattle genome, as discussed, enjoys a broad range of map resources that include: genetic linkage maps using microsatellite markers; markers comprising AFLP, EST, and BAC end sequences; a radiation hybrid map, and a BAC physical map [4, 5, 7, 9, 14–17]. Despite this, these resources fall a bit short in several ways. The genetic linkage and radiation hybrid maps lack sufficient levels of unambiguous markers, but, more troubling, the linkage map is a composite constructed across many separate bovine populations and thus doesn’t reflect a single bovine genome. The bovine BAC physical map is a composite map that was constructed from three different BAC libraries developed from three different cattle breeds (Hereford CHORI-240, Holstein RPCI-42, and Angus TAMBT) [4, 7]. Understandably, such haplotype and/or breed-specific variability in these map resources could translate into ambiguities evidenced by sequence-map comparisons, which may have impacted the fine-scale assembly, or previous validations of the bovine reference sequence.

We constructed a comprehensive optical map spanning the bovine genome, using genomic DNA from just one animal (L1 Dominette 014490; the same Hereford animal that was originally sequenced) in order to circumvent this array of issues. This new resource will provide the bovine community with a highly accurate and comprehensive physical map that enables direct and independent comparisons amongst sequence builds, with goals pointed at sequence finishing and discovery of genomic differences. Briefly, Optical Mapping is a single-molecule system that constructs high-resolution physical scaffolds, covering entire genomes to guide many stages of genome sequence assembly and validation [18–24]. Since it assembles genome-wide ordered restriction maps from massive datasets comprising randomly sheared genomic DNA molecules (~400 kb), artefacts associated with cloning and amplification are completely obviated. Furthermore, very long DNA molecules span complex genomic regions that are rife with repeats that generally hinder accurate sequence assembly without Optical Mapping analysis. As such, our optical map offers an uniquely effective means for resolving and mediating the differences between the two different bovine genome sequence assemblies in several ways: 1) recruiting new orphan sequence contigs that fill sequence gaps; 2) providing an independent resource that potentiates finishing through sequence gap characterization, and 3) enabling independent validations of sequence assemblies.

## Results

### Optical map dataset

Genomic DNA was prepared from L1 Dominette 014490 blood samples, after separation of white blood cells, and then BamHI restriction mapped using our Optical Mapping pipeline (Materials and Methods). This raw dataset holds 1,908,396 Rmaps (1 Rmap = 1 single molecule restriction map) ≥ 300 Kb, with an average size of 397.49 Kb (300–2,515.20 Kb) and a total mass of 758,574.97 Mb (~270 X coverage, before alignment, assuming a ~2.8 Gb genome). One Rmap is the restriction map of a single genomic DNA molecule; it represents the most fundamental unit of map data in functional ways akin to a sequence read. A second map dataset was contributed by Prof. Juan F. Medrano and after size filtering (≥300 kb) it added another 1,064,919 Rmaps, bringing the total raw dataset up to 2,973,315 (439 X coverage, before alignment).

### Initial evaluation of the genome builds UMD3.1 and Btau4.6 *via* pairwise alignments of the Rmap dataset

The UMD3.1 and Btau4.6 references were first evaluated for large-scale errors by inspection of the pairwise alignments [25, 26] of the entire Rmap dataset against BamH1 *in silico* restriction maps (constructed in the computer) of both sequence builds (Materials and Methods). These map *vs.* reference alignments produce files, similar to sequence SAM/BAM files, which note the location of each aligned Rmap (Additional file 1: Figure S1 and Additional file 2: Figure S2). Such alignments allow us to quickly filter-out marginal Rmaps from the raw dataset and provide an initial assessment of the completeness of a given sequence build [27]. The average Rmap coverage after alignment varies considerably between the two builds, with 42 X for Btau4.6, while UMD3.1 boasts 70 X. Additional file 1: Figure S1 and Additional file 2: Figure S2 also show a specific example of disparate rates of Rmap coverage, focusing on a 3.3 Mb region on chromosome 8, highlighted by a green box, where 8 Rmaps (~1 X coverage) are aligned to Btau4.6, compared to 527 Rmaps (~64 X) aligned to UMD3.1. Given these vastly different overall alignment rates and patterns, we chose UMD3.1 to serve as our reference sequence build for assembling the optical map.

### Optical map assembly

**Fig. 1:**
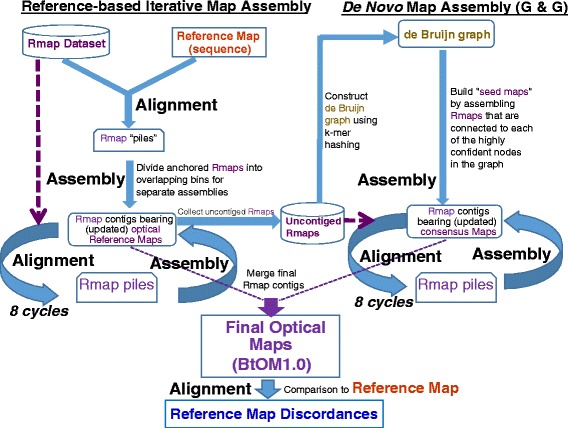
Strategy and workflow used for assembling the optical map-- BtOM1.0. Left side describes reference-based iterative map assembly: exhaustive pairwise alignments of the complete Rmap dataset (purple lettering) against the UMD3.1 sequence reference maps (red lettering) generated *in silico* produces “piles” of Rmaps. Such alignments are then divided into overlapping bins (1 Mb bins; 500 Kb overlap), which are then independently assembled into updated reference maps (optical contigs bearing consensus maps) that are used for 8 subsequent cycles (blue circular arrows) of alignment (entire Rmap dataset) and assembly, all performed without using the sequence reference. Right side depicts *de novo* map assembly, using those Rmaps from the entire dataset not recruited for optical map contig formation during iterative map assembly, which are used to construct a de Bruijn graph *via* k-mer hashing. “Seed” maps are then assembled from Rmaps in each each confident node within graph and used as an optical reference for pairwise alignments (piles) of Rmaps during iterative assembly (8 times; blue circular arrows). Bottom shows the merged assembly of updated optical contigs from each bin (iterative assembly) and contigs assembled from “seed” maps (*de novo* assembly) into the final optical map-- BtOM1.0, which was used to tabulate map *vs.* sequence discordances

Our optical map assembly strategy used a two-pronged approach involving iterative assembly, requiring a sequence reference [26], and *de novo* assembly for dealing with large-scale discordances (sequence *vs.* map) and gaps in the UMD3.1 build (Fig. 1). Many of these problematic regions are sparsely populated by Rmaps as evidenced from inspection of Additional file 1: Figure S1 (see regions highlighted by purple boxes). Accordingly, the workflow (Fig. 1) shows how iterative assembly selectively shunts uncontiged Rmaps, mostly originating from these problematic regions, into “Germinate and Grow” (G & G) for *de novo* assembly*.* Resulting optical map contigs from both sides of the workflow were then curated and combined for finishing the optical map. Details follow in the next two subsections.

#### Iterative assembly

We published a workflow in 2010 [26], termed “iterative assembly” (Fig. 1), which embedded genome assembly algorithms [28–31], originally designed to deal with small bacterial or fungal genomes, within a new pipeline. This pipeline supports the assembly and analysis of large mammalian and plant optical maps by distributing the computation into large numbers of independent jobs that can be executed on a high-throughput computing network. Briefly, iterative assembly uses an *in silico* restriction map of available genome sequence resources--contigs, scaffolds, pseudomolecules, etc.--as a reference for exhaustive pairwise alignment [25] of entire Rmap datasets. Both sequence data (UMD3.1) and actual genomic DNA molecules (Rmaps) are “cut” with the same restriction enzyme. Thusly placed Rmaps, termed “piles,” covering an entire genome, are then divided into 1 Mb overlapping bins along each chromosome for assembly; each bin is independently assembled into contigs. Each optical contig bears a consensus map, which now becomes the updated, independent reference; sequence information is no longer used in the assembly process. Repeatedly iterating this workflow increases optical contig length, number and depth.

Eight iteration cycles were performed using a BamH1 *in silico* map constructed from the UMD3.1 sequence build as the initial reference and with a minimum depth of 20 Rmaps. 3,048 contigs emerged after the first iteration ranging 404–2,943 Kb in size; averaging 1,826 Kb. However, after 8 iterations the number of contigs increased to 3,321, and their average span was boosted to 3,545 Kb (421–6,456 Kb). Contigs presenting very long tandem repeats were removed from this process. These 3,321 optical contigs were then grouped by chromosome, using alignments to UMD3.1 and each grouping was independently assembled into a total of just 79 contigs spanning 96.71 % of the UMD3.1 build.

#### de novo assembly

We have previously reported on the Map Assembler, a *de novo* optical map assembler capable of assembling bacterial maps [32]. However, the Map Assembler algorithm has polynomial complexity (degree >2) and exceeds feasible memory and time constraints for genomes of size >10 Mb. To face the challenge of assembling larger genomes, we’ve implemented Germinate and Grow (G & G), a new *de novo* assembly algorithm that will be fully described elsewhere. The conceptual basis for G & G is an extension of the de Bruijn graph approach to sequence assembly [33, 34]. Simply put, a whole genome optical map can be represented by the traversals of a certain graph, and the assembly problem is to discover those traversals from the input data set of Rmaps. Specifically, we use geometric k-mer hashing [35] to identify nodes in the de Bruijn graph that are very likely error-free and then traverse the “read” paths implied by the Rmaps containing instances of those nodes. This traversal allows us to localize the assembly; we then use the Map Assembler on the subsets of Rmaps that are near each other on this graph. We call the resulting consensus maps seed maps. The seed maps typically cover most of the genome and they reliably approximate highly confident paths in the graph.

The seed maps are then extended and refined using the iterative assembly engine (Fig. 1), producing another set of consensus maps. The error rate for these consensus maps is sufficiently low for resolving the corresponding Euler path and assembling all but the most repetitive regions of the genome. We then fill gaps in the assembly by repeating the process, generating another set of seed maps and extending and refining them. For this set of seed maps, we use a lower stringency (smaller value of k) and use only those Rmaps not already represented in the genome reference-based iterative assembly.

We used G & G to assemble just those Rmaps (2,448,748) that escaped assembly within the iterative assembly pipeline, which yielded 1,500 optical contigs, with most recapitulating those constructed by iterative assembly. As such, these *de novo* optical maps were largely used to augment and cross-validate optical map assemblies constructed by iterative assembly. The final bovine optical map--termed, “BtOM1.0”--comprises 78 contigs spanning of 2,575.30 Mb across the genome (alignments to UMD3.1 are found in Fig. 4), at an average depth of 77 Rmaps and an average contig size of 33.02 Mb (659.71 Kb–140.22 Mb; Table 1).

**Table 1:** BtOM1.0 contigs and their chromosome assignments

Optical map contig name	Chr. assigned	Contig size (Mb)	Ave frag size (Kb)	# of fragments	Pooled SD	# of contiged_Rmaps	Coverage (X)	Chr. end
BtOMcontig_0	chr1_OM	140.22	10.06	13,939	1.44	30,977	86.44	
BtOMcontig_1	chr2_OM	130.23	9.14	14,255	1.38	25,002	74.86	
BtOMcontig_2	chr6_OM	118.45	9.95	11,903	1.43	31,660	104.35	
BtOMcontig_3	chr4_OM	116.61	9.48	12,301	1.43	25,518	85.52	
BtOMcontig_4	chr11_OM	106.25	8.62	12,319	1.34	21,601	79.32	
BtOMcontig_5	chr3_OM	93.84	8.99	10,443	1.38	19,179	79.65	
BtOMcontig_6	chr7_OM	87.27	9.68	9,015	1.43	20,409	91.23	Rend
BtOMcontig_7	chr9_OM	84.26	10.48	8,036	1.47	21,152	98.28	
BtOMcontig_8	chr14_OM	79.81	9.30	8,580	1.37	19,868	96.42	Rend
BtOMcontig_9	chr10_OM	78.66	9.16	8,584	1.37	16,881	83.57	
BtOMcontig_10	chr5_OM	72.42	9.55	7,580	1.42	16,341	87.99	
BtOMcontig_11	chr20_OM	70.31	9.25	7,598	1.38	16,064	88.57	Rend
BtOMcontig_12	chr17_OM	69.22	8.87	7,802	1.38	15,249	85.40	
BtOMcontig_13	chr12_OM	68.49	10.06	6,806	1.44	17,380	99.26	
BtOMcontig_14	chr8_OM	67.78	9.44	7,180	1.38	16,346	93.45	
BtOMcontig_15	chr24_OM	61.70	8.83	6,985	1.34	13,714	85.83	
BtOMcontig_16	chr23_OM	51.93	8.12	6,397	1.27	11,238	83.85	Rend
BtOMcontig_17	chr21_OM	48.96	8.42	5,814	1.32	10,942	86.52	
BtOMcontig_18	chrX_OM	48.92	9.31	5,252	1.39	12,327	97.76	
BtOMcontig_19	chr22_OM	48.65	8.60	5,658	1.31	11,716	92.87	
BtOMcontig_20	chr26_OM	48.38	8.52	5,679	1.33	9,439	75.22	
BtOMcontig_21	chr27_OM	45.27	8.75	5,172	1.33	9,906	84.89	Lend
BtOMcontig_22	chr28_OM	42.83	8.66	4,948	1.32	8,814	79.69	
BtOMcontig_23	chr13_OM	42.25	7.24	5,838	1.19	7,245	65.93	Rend
BtOMcontig_24	chr8_OM	41.52	8.52	4,873	1.31	8,742	81.62	Rend
BtOMcontig_25	chr15_OM	41.17	8.51	4,838	1.16	12,747	120.55	
BtOMcontig_26	chr13_OM	40.80	8.65	4,716	1.31	10,335	98.34	
BtOMcontig_27	chr16_OM	40.45	9.14	4,426	1.38	9,294	89.46	
BtOMcontig_28	chr29_OM	38.81	8.50	4,564	1.20	8,285	82.48	
BtOMcontig_29	chr18_OM	37.17	7.77	4,784	1.25	6,532	67.64	
BtOMcontig_30	chr25_OM	35.31	7.07	4,994	1.16	5,605	60.68	
BtOMcontig_31	chr15_OM	33.58	9.29	3,615	1.38	7,351	85.37	
BtOMcontig_32	chrX_OM	31.48	10.26	3,068	1.47	8,223	101.92	
BtOMcontig_33	chr5_OM	30.96	9.21	3,361	1.39	6,918	87.43	
BtOMcontig_34	chr16_OM	26.17	8.47	3,089	1.29	5,361	79.51	Rend
BtOMcontig_35	chr19_OM	24.87	7.02	3,543	1.18	4,275	65.31	
BtOMcontig_36	chr10_OM	22.97	8.23	2,791	1.28	4,012	67.92	
BtOMcontig_37	chr21_OM	20.02	8.91	2,247	1.33	4,576	88.83	
BtOMcontig_38	chrX_OM	18.25	9.23	1,978	1.39	3,883	82.28	
BtOMcontig_39	chrX_OM	17.09	9.89	1,728	1.31	4,286	98.13	Lend
BtOMcontig_40	chr9_OM	17.02	8.49	2,004	1.29	3,479	79.17	Rend
BtOMcontig_41	chr19_OM	16.07	8.79	1,827	1.33	3,955	95.33	
BtOMcontig_42	chr12_OM	14.86	8.79	1,690	1.27	3,990	104.20	Rend
BtOMcontig_43	chr7_OM	14.62	6.60	2,214	1.16	2,335	61.06	
BtOMcontig_44	chr3_OM	13.22	7.30	1,812	1.14	2,100	61.76	
BtOMcontig_45	chr29_OM	12.61	6.83	1,845	1.06	1,845	55.85	Rend
BtOMcontig_46	chr1_OM	12.49	8.23	1,518	1.28	2,545	79.47	Rend
BtOMcontig_47	chrX_OM	12.13	8.23	1,474	1.27	2,933	93.12	Rend
BtOMcontig_48	chr18_OM	11.51	7.94	1,450	1.23	2,317	77.67	
BtOMcontig_49	chr5_OM	10.61	7.02	1,511	1.15	1,527	55.98	Rend
BtOMcontig_50	chr22_OM	10.42	7.10	1,467	1.20	2,247	82.47	Rend
BtOMcontig_51	chrX_OM	9.25	9.92	933	1.35	1,879	79.61	
BtOMcontig_52	chr19_OM	8.80	6.81	1,292	1.14	1,385	60.46	
BtOMcontig_53	chr3_OM	8.48	8.67	978	1.25	1,625	74.62	
BtOMcontig_54	chr25_OM	7.44	7.33	1,015	1.23	1,260	65.72	
BtOMcontig_55	chr15_OM	7.05	8.17	863	1.21	1,405	76.14	Rend
BtOMcontig_56	chr16_OM	6.81	8.19	832	1.26	1,304	74.28	
BtOMcontig_57	chr19_OM	6.62	7.18	922	1.18	1,280	75.50	Rend
BtOMcontig_58	chrX_OM	5.20	8.20	634	1.36	1,403	101.81	
BtOMcontig_59	chr7_OM	5.14	7.13	721	1.06	767	57.86	
BtOMcontig_60	chr16_OM	4.11	7.18	573	1.18	677	62.75	
BtOMcontig_61	chrX_OM	4.07	9.14	445	1.33	1,085	102.82	
BtOMcontig_62	chrX_OM	3.61	8.68	416	1.39	667	71.57	
BtOMcontig_63	chrX_OM	3.51	8.70	404	1.33	751	81.90	
BtOMcontig_64	chr19_OM	3.19	6.20	514	1.08	367	43.19	
BtOMcontig_65	chr5_OM	3.10	7.01	442	1.18	456	55.52	
BtOMcontig_66	chr28_OM	3.01	9.48	318	1.20	522	68.20	
BtOMcontig_67	chr17_OM	2.46	6.79	363	1.19	369	58.72	Rend
BtOMcontig_68	chr19_OM	2.39	6.75	354	1.11	359	57.08	
BtOMcontig_69	chr18_OM	2.37	6.96	341	1.13	367	59.18	Rend
BtOMcontig_70	chrX_OM	2.34	12.14	193	1.44	439	73.01	
BtOMcontig_71	chr18_OM	1.71	6.14	278	1.03	192	43.46	
BtOMcontig_72	chr14_OM	1.48	8.95	165	1.22	150	38.00	
BtOMcontig_73	chr16_OM	1.04	7.06	148	1.19	90	32.43	
BtOMcontig_74	chr18_OM	0.95	6.39	148	1.11	110	43.36	
BtOMcontig_75	chr14_OM	0.84	7.09	119	1.19	100	46.05	
BtOMcontig_76	chr18_OM	0.75	6.08	124	1.10	90	45.00	
BtOMcontig_77	chr18_OM	0.66	6.00	110	1.04	121	67.59	
Total/Ave.		2,575.30	8.91	289,156	1.28	567,886	77.00	

Note, Abbreviations: chr. = chromosome, PooledSD = Pooled standard deviation, AveFragSize = Average Fragment Size, and Ave. = average, Chr.end = chromosome end or telomere, Rend = right hand side of the contig is telomere, Lend = left hand side of the contig is telomere

**Fig. 2:**
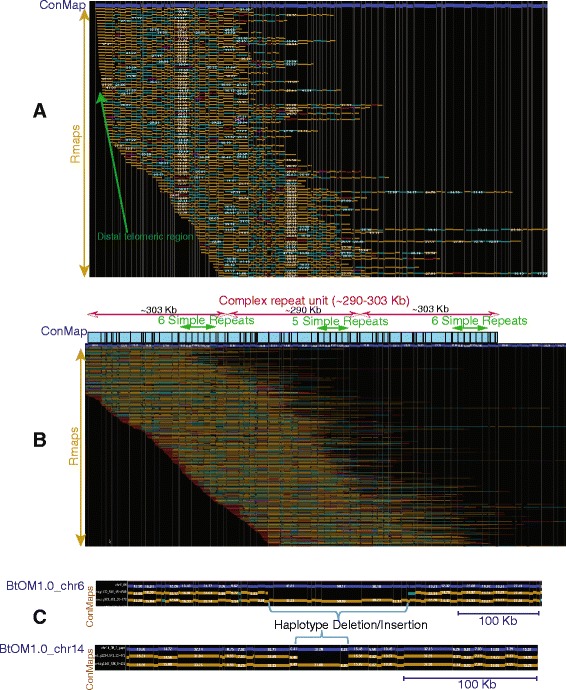
Examples of optical contigs, within BtOM1.0, reveal a telomeric region, complex tandem repeats and heterozygous structural variants. **a**: Telomeric regions are defined through assembly when Rmap contigs present sharply defined edges (green arrow); example shown is chr14, BtOMcontig_8 (Fig. 3); horizontal tracks depict Rmaps (single molecule restriction maps) with boxes representing individual restriction fragments, color keyed as: (gold—agreement; red—extra cut; cyan—missing cut; and purple—compound events) based on comparisons against the consensus map (ConMap; blue track). **b**: An optical map contig, showing the structure of a complex tandem repeat. **c**: Consensus maps of optical contigs (gold tracks) aligned to BtOM1.0 (blue track) showing heterozygous structural variants distinguished by deletions and insertions; numbers on each fragment bar show restriction fragment size (Kb). The first haplotype deletion/insertion is in the region corresponding to UMD3.1_chr6 62,804,591–62,981,784, and this region encoded an ATPase and an aminophospholipid transporter (APLT). The second case is corresponding to UMD3.1 chr14 35,756,042–35,808,353. There is a ~27 Kb missing sequence in UMD3.1 chr14 at this region, and the other part of this region encoded a solute carrier organic anion transporter

**Fig. 3:**
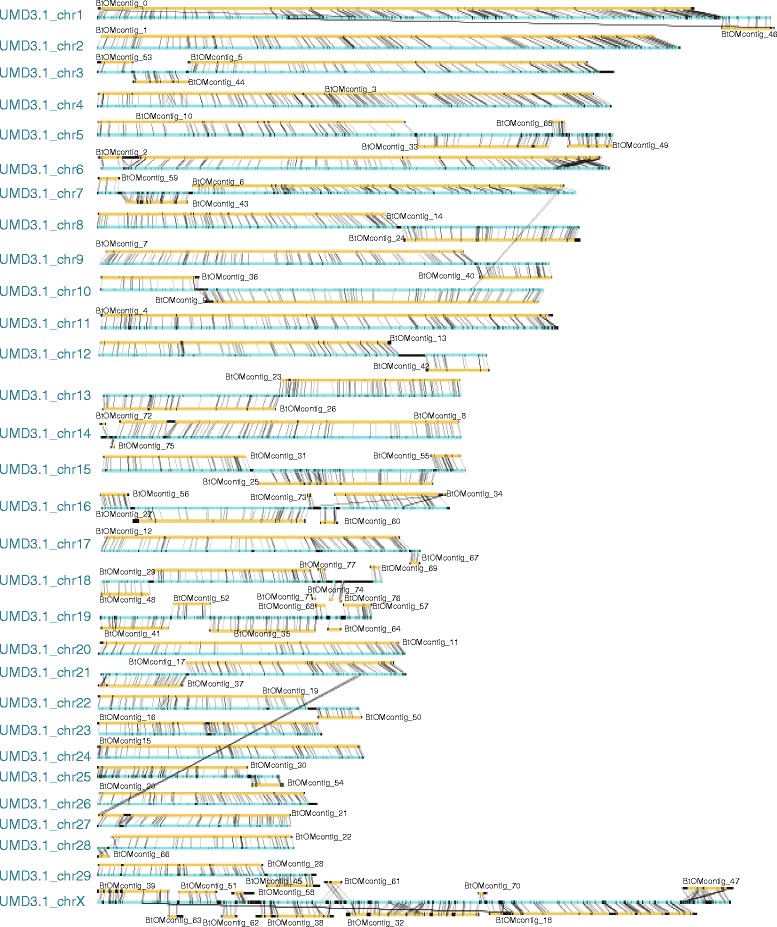
BtOM1.0 optical map (78 optical contigs) comprehensively aligned to UMD3.1. Optical contigs (consensus maps: BtOMcontig_0–77; gold tracks) extensively span across the UMD3.1 sequence assembly (cyan tracks) revealing a minimal number of gaps in the optical map (BtOM1.0)

The haploid bovine genome harbors 29 acrocentric, autosomal chromosomes, and one sex chromosome, or 60 telomeric ends. Accordingly, 20/78 optical contigs (BtOMcontig_6, 8, 11, 16, 21, 23, 24, 34, 39, 40, 42, 45, 46, 47, 49, 50, 55, 57, 67, 69) present sharply demarcated ends (Figs. 2a and 3; Tables 1 and 2), which indicate that they’ve spanned into the repetitive sequences near telomeres. The remaining 40/60 chromosome ends are not, or, are partially spanned by optical maps because the short arms of these acrocentric chromosomes are densely populated by repeats, making them intractable to our analysis. Interestingly, we find that BtOMcontig_4 has ~6 Kb tandem repeats at one end, which also shows alignment to chromosome 11. In addition, BtOMcontig_2 presents tandem repeats with a repeat unit consisting of multiple BamHI fragments with a total unit mass of ~290 Kb and is anchored on bovine chromosome 6 (Fig. 2b; Table 1). Lastly, an additional 5 optical map contigs, spanning 19.43 Mb show evidence of heterozygosity, manifested as indels (40 Kb, 173 kb, 248 Kb, 348 Kb, and 418 Kb) on chromosomes (6, 14, 15, and X) within BtOM1.0 as illustrated by the examples in Fig. 2c. All the contiged Rmaps for each chromosome, and all the optical map contig consensus maps are available at GitHub (https://github.com/schwartz-lab/BovineGenomeOMdata/)

**Table 2:** Alignment statistics for BtOM1.0 *vs.* UMD3.1 and Btau4.6

Chr. No	Chr. size (Mb)		No. of aligned map segments		Ave. aligned map segment size (MB)		Total mass of aligned map segment (Mb)		% Coverage		Telomere extension by OM (kb)			
	UMD3.1	Btau4.6	UMD3.1	Btau4.6	UMD3.1	Btau4.6	UMD3.1	Btau4.6	UMD3.1	Btau4.6	UMD3.1		Btau4.6	
1	158.34	161.43	5	8	27.92	15.79	139.62	126.3	88.18	78.24	646	392	668	17
2	137.06	141.97	2	6	60.62	18.95	121.23	113.72	88.45	80.10	123	0	135	117
3	121.43	126.84	3	10	34.80	9.83	104.41	98.33	85.98	77.52	726	0	300	0
4	120.83	123.81	5	4	21.32	24.88	106.58	99.53	88.21	80.39	255	0	397	0
5	121.19	125.25	7	6	15.23	16.34	106.64	98.04	87.99	78.28	256	567	345	1024
6	119.46	122.52	3	5	34.84	18.58	104.52	92.88	87.49	75.81	535	396	632	2132
7	112.64	113.03	5	9	18.89	9.62	94.45	86.54	83.85	76.56	173	125	58	718
8	113.38	116.85	3	4	32.64	22.60	97.92	90.39	86.36	77.36	388	1682	514	1275
9	105.71	108.5	2	4	46.94	21.40	93.88	85.58	88.81	78.88	276	61	1002	152
10	104.31	105.98	7	8	14.22	10.58	99.52	84.66	95.41	79.88	0	391	402	298
11	107.31	109.99	2	11	47.66	8.10	95.31	89.15	88.82	81.05	436	631	402	1019
12	91.16	85.12	2	6	38.06	11.31	76.11	67.85	83.49	79.71	177	52	1938	46
13	84.24	84.21	2	4	37.09	17.34	74.17	69.34	88.05	82.34	316	58	290	72
14	84.65	81.22	3	5	24.44	13.31	73.32	66.53	86.62	81.91	0	72	844	89
15	85.3	84.47	5	8	14.49	7.90	72.45	63.17	84.94	74.78	302	42	1240	249
16	81.72	77.71	9	8	7.57	7.45	68.15	59.57	83.39	76.66	0	385	637	99
17	75.16	76.28	2	3	32.56	19.77	65.12	59.31	86.64	77.75	254	39	265	71
18	66	65.81	7	7	6.88	6.09	48.15	42.6	72.95	64.73	252	59	883	59
19	64.06	64.85	6	8	8.96	6.34	53.77	50.68	83.94	78.15	188	147	1140	101
20	72.04	75.69	1	2	63.53	29.96	63.53	59.92	88.19	79.17	694	27	529	173
21	71.6	69.08	4	5	15.54	10.86	62.17	54.29	86.83	78.59	407	0	2080	1248
22	61.44	61.6	3	4	17.80	12.20	53.4	48.78	86.91	79.19	625	155	931	1050
23	52.53	52.33	2	3	22.83	13.98	45.65	41.94	86.90	80.15	410	0	0	264
24	62.71	64.51	1	5	56.86	10.54	56.86	52.72	90.67	81.72	250	96	862	92
25	42.9	44.08	2	4	18.16	8.47	36.31	33.89	84.64	76.88	295	367	0	412
26	51.68	51.83	1	2	43.31	18.89	43.31	37.78	83.80	72.89	370	0	389	0
27	45.41	48.46	1	2	38.83	18.52	38.83	37.03	85.51	76.41	976	109	145	336
28	46.31	45.96	4	4	10.27	9.30	41.09	37.18	88.73	80.90	809	462	809	514
29	51.51	51.81	3	2	14.34	19.57	43.02	39.13	83.52	75.53	192	164	583	770
X	148.82	88.65	33	31	3.56	2.19	117.59	67.91	79.01	76.60	0	2327	4680	2845
Total/Ave.	2660.90	2629.84	135	188	*17.02*	*10.93*	2297.08	2054.74	86.33	78.13	19135		38346	

### Construction of chromosome-wide optical maps and their comparison to UMD3.1 and Btau4.6

**Fig. 4:**
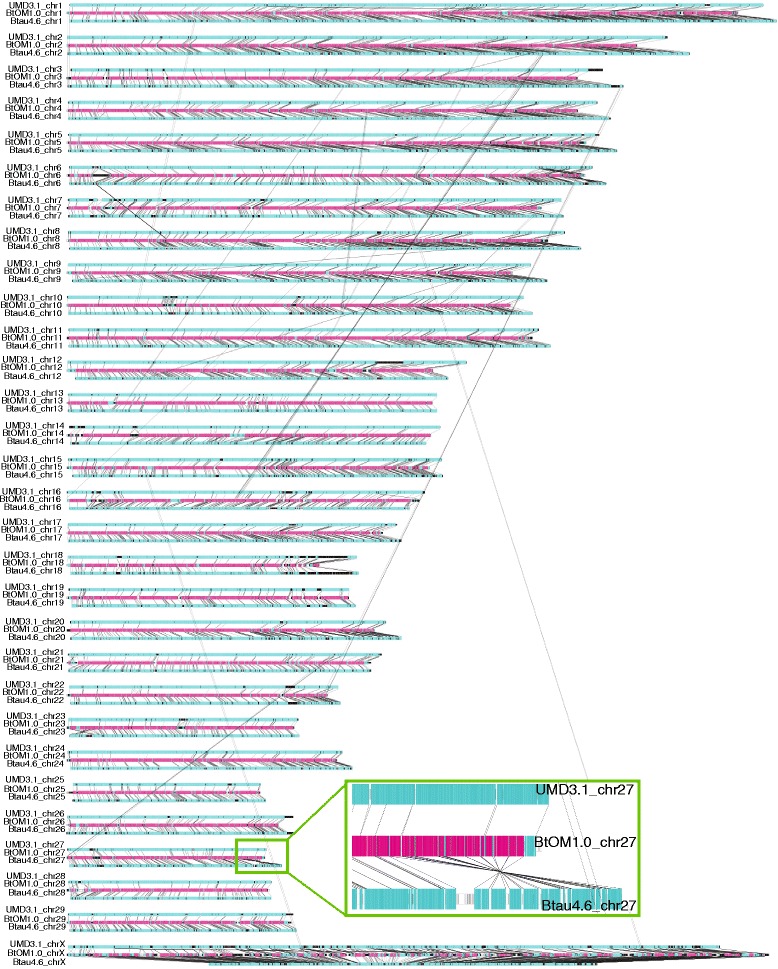
Genome-wide alignment of BtOM1.0 to UMD3.1 and Btau4.6 sequence assemblies. Tracks show BtOM1.0 (center) alignments to UMD3.1 (top) and Btau4.6 (bottom) for each chromosome. Red highlights (center track) restriction fragments aligned to both *in silico* maps of UMD3.1 and Btau4.6; cyan (center track) highlights alignment of BtOM1.0 to UMD3.1, or Btau4.6; and white or black highlights no alignments to either sequence build. Inset (green box) shows a zoomed view of chr27 detailing a large-scale discordance with the optical map: an inverted sequence assembly within Btau4.6. Also note transposed sequence assemblies, flagged by black lines running between separate chromosomes

**Fig. 5:**
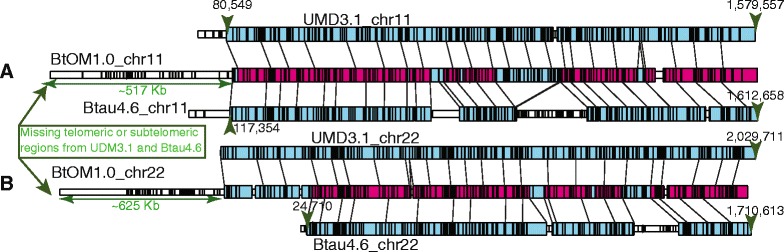
Examples of optical map coverage within telomeric, or sub-telomeric regions. **a**: BtOM1.0_chr11 aligned to chr11 of both UMD3.1 and Btau4.6. Lines show BamHI restriction sites with red highlighting those BtOM1.0_chr11 track (center) restriction fragments aligned to both UMD3.1_chr11 and Btau4.6_chr11. White highlights unaligned BtOM1.0_chr11 restriction fragments and extend 517 kb past both the UMMD3.1_chr11 and Btau4.6_chr11 sequence. **b**: BtOM1.0_chr22 aligned to chr22 of both UMD3.1 and Btau4.6. (Same color scheme as **a**.) The white region on BtOM1.0_chr22, is unaligned to both sequence builds and extends ~625 kb past UMD3.1_chr22

The optical contigs generated by iterative and *de novo* map assembly (Fig. 1) were merged through assembly of their consensus maps into 78 final optical contigs. They were then ordered and oriented, through alignment against a BamHI *in silico* restriction map constructed from the UMD3.1 sequence build (Fig. 3). Chromosome-wide optical maps, BtOM1.0_chr1-29 and BtOM1.0_chrX, were constructed with 500 Kb gaps inserted between any two of optical map contigs anchored on the UMD3.1 sequence assembly (BtOM1.0 available at GitHub: https://github.com/schwartz-lab/BovineGenomeOMdata/). This workflow constructed 30 chromosome-wide optical maps that were aligned to both Btau4.6 and UMD3.1 sequences using local alignment (Fig. 4). A series of contiguous restriction fragments that align between BtOM1.0 and the *in silico* maps of a sequence build is called a “map segment.” Tabulations describing these aligned map segments are listed in Table 2. In total, 135 map segments (over 78 optical contigs) present a total of aligned sequence segment mass of 2,297.08 Mb, with an average size of 17.02 Mb, or ~86 % of UMD3.1 are covered by optical maps. Map coverage of UMD3.1 ranges from 73 to 95 %. 50/60 chromosome ends, 19.14 Mb in total, within UMD3.1, are extended by optical maps (Fig. 5; Tables 1 and 2). For Btau4.6, 188 map segments align to BtOM1.0, with a total mass of 2,054.74 Mb (~78 % of Btau4.6) and an aligned map segment size averaging 10.93 Mb. The optical map coverage of Btau4.6 by optical maps is less than that tabulated for UMD3.1 and it ranges from ~65 to 82 % for all 30 bovine chromosomes. Lastly, 55/60 of the Btau4.6 chromosome ends are extended by optical maps with a mass totalling 38.35 Mb (Table 2).

### Discordance calling between optical maps and sequence assemblies

**Fig. 6:**
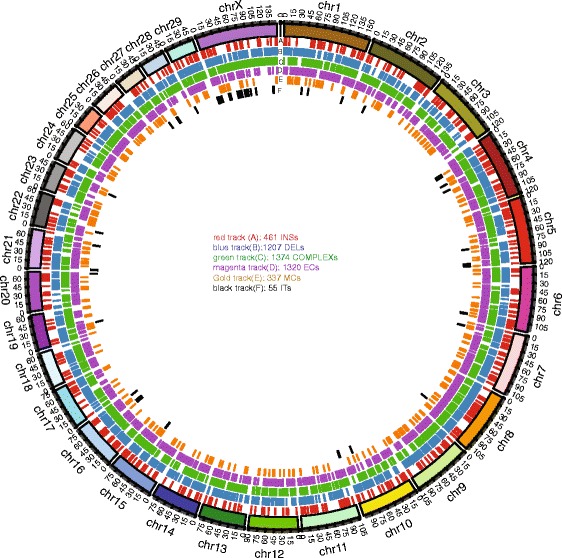
Circos plot of discordances found between BtOM1.0 (optical map) and UMD3.1 (sequence assembly). The tracks A, B, C, D, E, and F represents the six categories of discordances: Insertion (INS), implying missing sequence; deletions (DELs) implying extra sequence; compound multiple events including insertion/deletion/inversion etc. (COMPLEX); missing restriction sites (MCs); extra restriction sites (ECs); and large inversions or translocations (ITs)

**Fig. 7:**
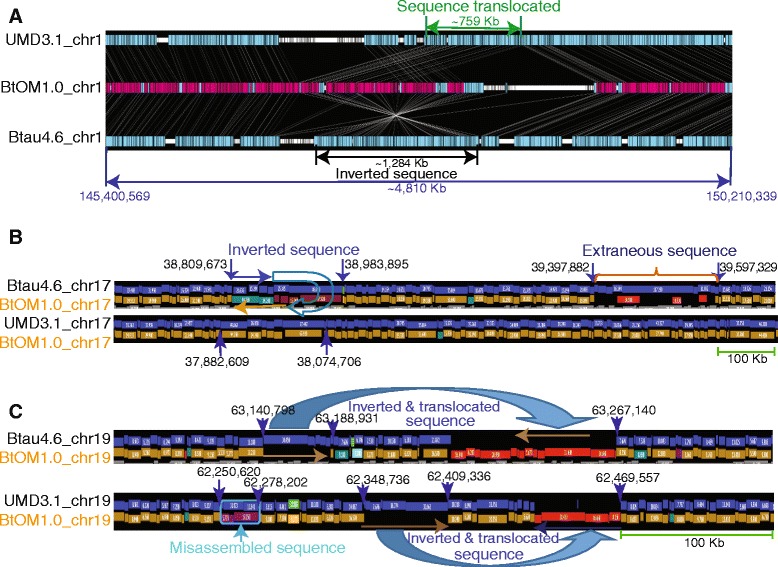
Examples of large-scale map *vs.* sequence discordances (IT discordances). **a**: GPS (Methods) viewer shows the BtOM1.0_chr1 optical map (center track) alignments to UMD3.1_chr1 and Btau4.6_chr1 revealing 759 Kb translocated region (UMD3.1) and a 1,284 Kb inversion (Btau4.6). Color scheme is the same as Fig. 5, with cyan highlighting BtOM1.0_chr1 portions aligning to UMD3.1_chr1 *or* Btu4.6_chr1. **b**: ~1 Mb region of chromosome 17: the *in silico* maps (blue track) of UMD3.1_chr17 perfectly align to the BtOM1.0_chr17, but Btau4.6_chr17/BtOM1.0_chr17 alignment reveals two misassembled regions in the Btau4.6 sequence build: an inversion (blue and gold arrows) and extraneous sequence. Restriction fragments (undulating boxes) are color keyed as: gold (agreement with blue track, or reference); red (extra cuts in optical map); cyan (missing cuts in optical map); and purple (compound events). **c**: ~500 Kb region on chromosome 19: alignment of BtOM1.0_chr19 and Btau4.6_chr19 (blue track) suggest that the Btau4.6_chr19 sequence (blue arrow) was probably misplaced and should be inverted and placed nearby. BtOM1.0_chr19 and UMD3.1_chr19 (blue track) alignment suggests that sequence here also presents misassemblies similar to Btau4.6_chr19

Discordances between BtOM1.0 and the UMD3.1 were called based on the alignments between the consensus maps that were trimmed and stripped off from the last (8^th^) cycle of iterative assembly (Fig. 1; Methods; [26]) and then manually curated. Complex discordances required directed alignment and assembly steps, complemented by additional curation, for their complete characterization. There are, in total, 4,754 discordances called between BtOM1.0 and the UMD3.1 based on only confident alignments and these discordances are tabulated as **six** categories (Additional file 3: Table S1; Table 3; Figs. 5 and 6): ***(1)*** large segments of inverted/translocated sequence (55; involving 31.11 Mb sequencing; Fig. 7a), ***(2)*** COMPLEX-events/misassembly/inversion (1,374; involving 111.54 Mb sequences; Fig. 7b and c), ***(3)*** INS-insertion/missing sequence (461;involving 15.38 Mb sequences), ***(4)*** DEL-deletion/extra sequence included (1,207; involving 44.82 Mb sequences), ***(5)*** EC-extra restriction site (1,320), and (***6)*** MC-missing restriction site (337).

**Table 3:** Statistics for the six categories of discordances between sequence builds and BtOM1.0

Statistics for the discordances between sequence builds and BtOM1.0							
Sequence build	Complex	IT	DEL	INS	EC	MC	Total
UMD3.1	1374	55	1207	461	1320	337	4754
Btau4.6	2331	102	2596	782	1166	486	7463

Similarly, Btau4.6 discordances were tabulated as just described for UMD3.1, but relied on the same optical consensus maps created from UMD3.1. These efforts identified 7,463 discordances in the Btau4.6 sequence assembly (Additional file 4: Table S2; Table 3; Additional file 5: Figure S3). Such tabulations include 102 large segments of inverted/translocated (IT) (involving 61.65 Mb; Fig. 7a), 2,331 COMPLEX-complex events/misassembly/inversion (involving 273.14 Mb; i.e., Fig. 7b and c), 782 INS-insertion/missing sequences (involving 82.71 Mb - sequence), 2596 DEL-deletion/extraneous sequence (involving 99.48 Mb), 1,166 EC-extra restriction sites, and 486 MC-missing restriction sites.

## Discussion and Conclusions

A whole genome optical map, BtOM1.0, of the *B. taurus* Hereford breed, L1 Dominette 01449 was constructed using the same animal employed for whole genome shotgun sequencing, which was also the daughter of the Hereford bull L1 Domino (registration number 41170496) used for the construction of the previously analyzed BAC library [4]. The optical map spans 2,575.30 Mb across the *B. taurus* genome and comprises 78 optical contigs, which provide accurate size estimations for 289,155 BamHI restriction fragments. Alignments between BtOM1.0 and *in silico* restriction maps of UMD3.1 and Btau4.6 revealed numerous discordances at genomic length scales reaching from a restriction site to portions of a chromosomal arm. On average, there is a BamHI site every 8.91 Kb, and such “marker” density is far greater than the bovine genetic map (∼7,000 markers [14]) and the composite map (∼17,000 markers) which combines linkage and radiation hybrid resources [8, 9]. The size of the *B. taurus* genome, estimated by Optical Mapping, is similar to those estimates provided by the UMD3.1 and Btau4.6 genome sequence assemblies (2,660.90 Mb and 2,629.84 Mb respectively), but is ~17 % smaller than the 3,088 Mb size estimated by BAC fingerprinting [4]. Genome size differences may stem, in part, from genome analysis efforts that employed DNA samples from three different cattle breeds: Hereford, Holstein and Angus [4], whose separate BAC ibraries were used for previous mapping efforts.

Our analysis, through alignments to BtOM1.0, showed that Btau4.6 presented far more discordances as compared to UMD3.1. Overall, we found that Btau4.6 presented almost double the number of discordances across most of its 6 categories of sequence *vs.* map discrepancies. They include COMPLEX (misassembly) discordances as compared to UMD3.1 (2,331 vs. 1,374), which more than doubled the amount of affected sequence (273.14 Mb *vs.* 111.54 Mb). Further comparisons of Btau4.6 to UMD3.1 also showed a doubled count of DELs (extra sequence) discordances (2,596 vs. 1,207) and the amount of affected sequence (99.48 Mb *vs.* 44.81 Mb). Similarly, Btau4.6 presented a doubled rate of called INS (missing sequence) and Inverted/Translocated discordances (Table 3).

**Fig. 8:**
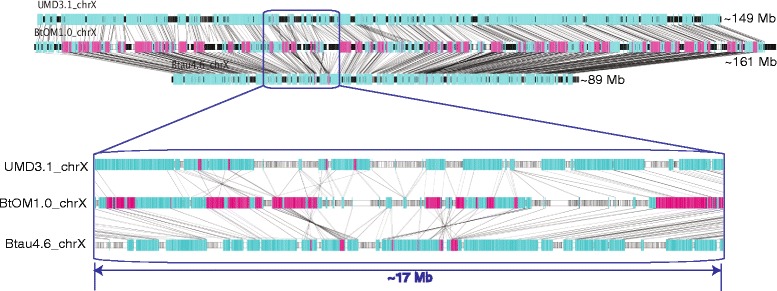
Map alignments among BtOM1.0 chromosome X and UMD3.1 and Btau4.6. GPS (Methods) (color scheme as in Figs. 3 and 5) reveals numerous and very large-scale discordances as a series of misassemblies. Zoomed portion shows details of such misassembled regions

Comparisons of Btau4.6 and UMD3.1 to BtOM1.0 also revealed large-scale difference between these sequence assemblies. Such issues became most apparent through our analysis of optical map alignments at telomeric regions, or ends of chromosomes. Table 2 shows that Btau4.6 is missing more sequence at chromosome ends (38.35 Mb**)** as compared to UMD3.1 (19.14 Mb). Also, the Btau4.6 assembly of the X chromosome excluded ~60 Mb of sequence relative to BtOM1.0 (Fig. 8). Therefore, our comparative analysis results of UMD3.1 and Btau4.6 based on alignments to BtOM1.0 are in line with previous reports [11, 12], and affirm the NCBI’s current designation of UMD3.1 sequence assembly as the “reference” assembly and the Btau4.6.1 assembly as the “alternate” assembly [36].

There are numerous sequence gaps in the two *B. taurus* genome sequence assemblies (74,425 in UMD3.1 and 66,276 in Btau4.6). However, most of the sequence gaps are small, in that there are only 606 sequence gaps ≥ 2 kb in UMD3.1 and 5,450 in Btau4.6. Importantly, greater than 96 % of these gaps in UMD3.1 and Btau4.6 were successfully bridged by BtOM1.0 (584 and 5401, respectively). Accordingly, this analysis begs the question: Are the discordances called in UMD3.1 and Btau4.6, through alignments to BtOM1.0, largely due to sequence gaps inserted into the two sequence assemblies? We explored this question by intersecting the sequence gap and discordance coordinates from both sequence assemblies, and identified within UMD3.1 only 162/4,574 discordances: (86 COMPLEXs, 46 DELs, 5 INSs, 15 ECs, 0 MC, and 10 Inverted/Translocated), which intersected just 167/606 sequence gaps (≥2 kb). For Btau4.6 we found 3,801/7,463: (1,720 COMPLEXs, 1,625 DELs, 254 INSs, 160 ECs, 2 MCs, and 40 ITs; Materials and Methods), which intersected 4,586/5,450 sequence gaps (≥2 Kb). Thus, 27.6 % of the large sequence gaps in UMD3.1 contribute to only 3.4 % of the discordances called in UMD3.1, while 84.2 % of the large sequence gaps in Btau4.6 are responsible for 50.8 % of the called discordances in this assembly. As such, this simple analysis further substantiates the superior quality of UMD3.1 *vs.* Btau4.6, which in part, is reflected by the high rate of parsimoniously inserted sequence gaps.

**Fig. 9:**
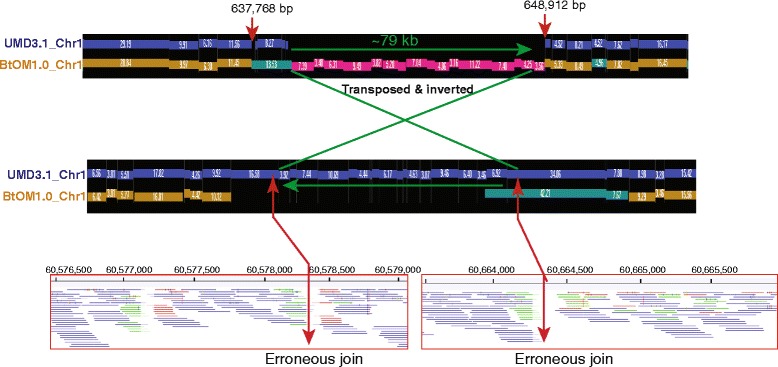
Optical map (BtOM1.0_chr1) reveals a 79 Kb sequence assembly as being transposed and inverted in UMD3.1_chr1. (Top) Alignment shows an unaligned 79 Kb segment (pink bars) within the optical map and a corresponding gap in the sequence. (Middle) Same 79 Kb segment within the UMD3.1 build but apparently transposed to 60,578,754 bp and inverted. Accordingly, there is a 79 Kb gap in the optical map. (Bottom) Illumina paired-end Dominette L1 sequence data, aligned to UMD3.1 corroborates sequence misjoining points (red arrows) at 60,578,000 bp and 60,664,500 bp. PCR experiments confirms that the 79 Kb segment should be placed between 637,768 and 648,912 on chromosome 1 (data not shown). Paired-end reads mapped to UMD3.1 showing correct orientations with both ends mapped are named an intact pair (blue tracks). When only one end is mapped, or mapping shows wrong orientation, or revealing discordant distances between mapped read pairs, these events are termed broken pairs. Reads of a broken pair that map to a unique location against the reference are colored green or red, according to whether they mapped in the forward, or reverse, orientation respectively

Our systematic tabulation and curation of discordances found through comparison of BtOM1.0 *vs.* UMD3.1, or Btau4.6 will greatly facilitate future improvements of *B. taurus* genome sequence assemblies in order to build a more accurate and unified version of the reference sequence. Because BtOM1.0 was constructed from DNA derived from the very same animal that was sequenced, this physical map provides direct comparisons to these other resources that are not affected by genotype differences manifested by other breeds, or even animals of the same breed. Although there are many map resources available for the *B. taurus* genome, which include genetic linkage maps [5, 6, 14–17, 37, 38], radiation hybrid maps [8, 9, 39], BAC physical maps [4, 7], cytogenetic maps [40, 41] and comparative maps between cattle and human [10, 42, 43], the resolution of these maps can be modest. Consider that the *B. taurus* composite map of integrated linkage/radiation hybrid maps [9, 39] and BAC physical maps [4, 7] features the greatest number of markers (17,254 markers), but with a density of only ~180 kb/marker. In comparison, BtOM1.0 boasts an average restriction site density of 8.91 Kb, which fostered resolution of difficult-to-discern errors in sequence assembly. For example, Fig. 9 shows a 79 Kb region that was inverted and misplaced based on alignment to BtOM1.0, which was also substantiated by new sequence data and PCR.

**Fig. 10:**
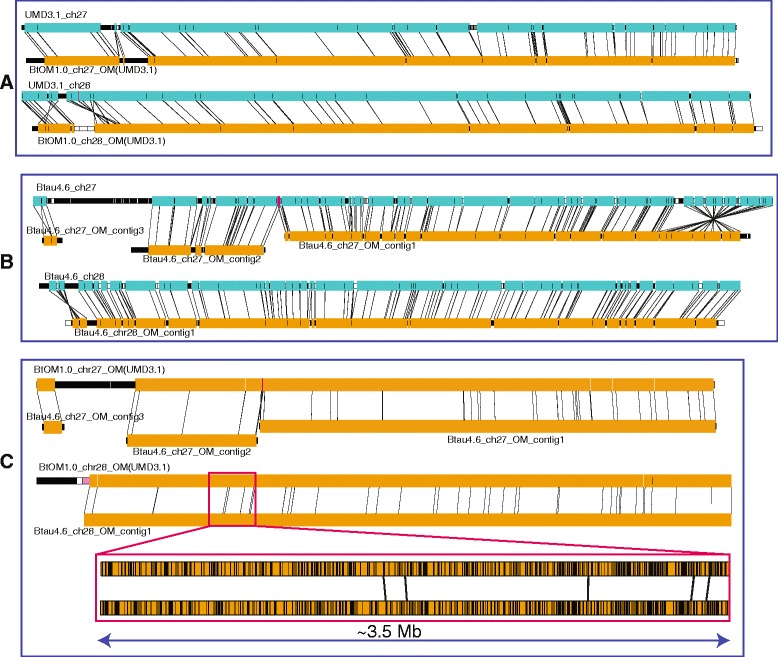
Comparisons of optical maps assemblies seeded by UMD3.1, or Btau4.6. (*Cyan tracks show in silico restriction maps generated from sequence; Orange tracks show optical maps. Striated black, or white-filled boxes flag unaligned map fragment(s). Black vertical lines demarcate restriction fragments, or map alignments.)* **a**: Alignments of BtOM1.0_chrs 27, 28, against the *in silico* maps of UMD3.1--chrs 27 and 28 (also see Fig. 3). **b**: Iterative assembly results for chrs 27 and 28 using Btau4.6 as the reference sequence. Note large inversion (far right), not present in UMD3.1, or optical maps, revealed within the Btau4.6 chr27 sequence. **c**: Alignment of the optical maps shown in (**a**) and (**b**). Inset shows 3.5 Mb region; vertical lines locate BamHI restriction sites. Pink fragment (500.0 Kb) shows the gap between the two optical map contigs in BtOM1.0 chromosome 28

During the course of writing this manuscript, a reviewer questioned the extent of map errors and possible biases that may be introduced through our selective use of UMD3.1 as the reference genome for BtOM1.0. Although our previous publications report a high degree of accuracy and minimal biases stemming from the iterative assembly pipeline [22, 23, 26], we compared the iterative assembly of optical maps constructed from UMD3.1 *vs*. Btau4.6 using chromosomes 27 and 28 (Fig. 10)*.* Using Btau4.6 as the reference sequence, eight iterations (Fig. 1) and merging of optical contigs produced three optical map contigs for chromosome 27 and a single optical map contig was derived for chromosome 28. Alignments show that these new maps are essentially identical to BtOM1.0 except for a few restriction site differences (2 extra cuts, 5 missing cuts for chromosome 27; 4 missing cuts for chromosome 28). We attribute these minor differences to heterozygosity, since our calling of discordances uses a single representation of the physical map created by Optical Mapping. However, over an entire chromosome multi-Mb-scale differences are apparent. Fig. 10b shows three optical map contigs aligned to Btau4.6, chromosome 27, presenting a large gap (~5.3 Mb) between contig2 (8.80 Mb) and contig3 (1.89 Mb), and no gap between contig1 (30.52 Mb) and contig2, relative to BtOM1.0; while a single contig (45.27 Mb; Figs. 3 and 10) spans the same chromosome using UMD3.1 chromosome 27. In comparison, chromosome 28 shows a single optical map contig (43.20 Mb) generated from the Btau4.6 sequence as the starting reference, while two optical map contigs (contig1, 42.84 Mb; contig2, 3.01 Mb; Figs. 3 and 10) formed using the UMD3.1 sequence for chromosome 28 (Fig. 10a). The absence of the small contig2 (3.01 Mb) from the Btau4.6 derived optical contigs implies reduced coverage for chromosome 28. As such, our analysis shows that BtOM1.0 bears minimal *local* biases stemming from the choice of sequence build used for iterative assembly, but the overall optical map coverage varies. Fortunately, absent, or problematic genomic regions would then be covered, as required, by optical maps constructed by *de novo* techniques (Fig. 1). Consequently, the need for *de novo* assembly steps is minimized by judicious selection of a reference genome for iterative assembly of an optical map.

We conclude that BtOM1.0 will prove to be a valuable resource for advancing the state of current sequence assemblies, by serving as a largely independent physical scaffold, as shown in Figs. 5, 6, 7, 8, 9 and 10, but perhaps, more importantly, as a platform to support future comparative studies, focusing on structural variation amongst different cattle breeds, or within populations. Lastly, errors always accompany any ambitious effort pointed at comprehensive analysis of entire genomes. Accordingly, the true merits and accuracy of a new resource, such as BtOM1.0, will be comprehensively assessed over time by individual researchers in the bovine community.

## Methods

### DNA sample preparation

DNA was extracted from a peripheral blood sample (Dominette L1 014490; American Hereford Association registration number 42190680) provided by Leeson J. Alexander from Fort Keogh Livestock and Range Research Laboratory of USDA Agricultural Research Service; Miles City, Montana. Blood cells were pelleted at 350 g for 10 min at 4 °C and resuspended in red blood cell lysis solution (0.8 % NH_4_Cl, 0.1 mM EDTA, pH = 7.4) at room temperature for 10 min; white blood cells were then pelleted at 350 g at 4 °C for 10 min and then resuspended and washed in Dulbecco’s PBS (1.54 mM KH_2_PO_4_, 155.17 mM NaCl, 2.71 mM Na_2_HPO_4_, pH 7.2). High Molecular Weight (HMW) DNA for Optical Mapping was obtained by suspending white blood cells in 1 % low-melting point agarose in distilled water to form gel inserts [44] (1 million cells/ml) and lysed in modified NDSK (1 mg/ml proteinase K, 1 % lauroylsarcosine, 0.5 M EDTA, 1 M NaCl, pH 8.0) at 50 °C for two overnights with one switch of fresh NDSK solution after the first overnight; HMW DNA was extracted from prepared inserts for optical mapping as previously described [20, 24, 45].

### Optical mapping

Optical mapping surfaces were prepared as previously described [19, 21, 45, 46]. Briefly, glass cover slips (22 × 22 mm, Fisher’s Finest) were cleaned by boiling in Nano-Strip (Cyantek Corp., Freemont, CA), followed by boiling in concentrated HCl, extensively rinsed with high purity water and sonicated until the pH of the wash reached 6.0 within 30 min, and then washed with ethanol twice with sonication. Cleaned glass cover slips were derivatized using trimethyl silane: (N-trimethoxysilypropyl-N,N,N-trimethylammonium chloride; 130 μl) and vinyl silane: (vinyltris(trimethysiloxy)silane); 15 μl) in 250 ml in distilled water to confer a positive charge and provide chemical moieties for covalent bonding of the acrylamide overlay to the surface.

### DNA mapping, image acquisition, and processing

Bovine genomic DNA molecules (~400–500 kb) were premixed with lambda DASH II bacteriophage DNA (Stratagene, La Jolla, CA) as an internal sizing standard and then deposited on optical mapping surfaces using a silicone microchannel device [24]. A fully automated image acquisition microscope workstation (GenomeZephyr) with Mightex LED illumination (San Francisco, CA) acquired image data that was automatically processed by machine vision, within a pipeline, which compiled large files comprising ordered restriction maps for each imaged molecule (Rmap) [24].

### Optical map assembly

Previous work [22] had confirmed that iterative assembly, which relies on a sequence reference map, constructs unbiased optical maps that are essentially equivalent to those crafted by a *de novo* method using a “divide and conquer” approach [22, 23, 26, 46]. Iterative assembly simply uses the reference sequence for anchoring Rmaps, which are then independently assembled in to optical contigs (Fig. 1). These newly assembled optical contigs become the updated reference for 8 cycles of alignment and assembly, which increase their breadth and depth. All accomplished without use of the sequence reference map. Accordingly, if a sequence reference suffers from many misassemblies, or gaps, *de novo* approaches are used to assemble across such regions. Because of sequence assembly issues, the *B. taurus* optical map incorporates these two assembly strategies for efficient and comprehensive map assembly, which used our G & G algorithm for *de novo* assembly.

We first used reference-based iterative map assembly and then removed the Rmaps in these assembled optical map contigs from the whole Rmap dataset, the leftover or the uncontiged Rmaps were for *de novo* map assembly *via* G & G. The combined map assembly strategy (Fig. 1) ensured the completeness of the final optical maps by maximizing the recovery of optical contigs from genomic regions not covered, or from heavily misassembled sections in the reference maps (UMD3.1 and Btau4.6).

### Construction of chromosome-wide maps

Eight cycles of iterative assembly, using the UMD3.1 *in silico* map as the reference, produced thousands of overlapping optical contigs. Consensus maps of these contigs were merged using the map assembler into large-scale optical maps (Fig. 3). These large-scale optical maps were then further augmented and refined through additional merging operations using optical consensus maps generated from *de novo* assembly. After alignment to the UMD3.1 *in silico* BamHI restriction map, they were manually joined into chromosome-wide optical maps and viewed using GnomSpace --a map- centric genome viewer that facilitates inspection of alignments.

### Calling discordances between the *in silico* maps of sequence assemblies and optical maps

As previously described [26] the iterative assembly pipeline automatically calls discordances, or structural variants using a reference map (UMD3.1, or Btau4.6. 5) classified as: ***(1)*** missing restriction sites [MC], ***(2)*** extra restriction sites [EC], ***(3)*** missing sequences, or gaps [DEL], ***(4)*** extra sequences [INS], and ***(5)*** compound, or complex [COMPLEX].

Very large scale, or complex discordances involving apparent translocations of sequence assemblies between chromosomes required manual intervention. These discordances were flagged as ITs (Inverted or Translocated sequences) and curated using map viewers developed in our group: GPS (unpublished work) and GnomSpace [26].

### Genome viewer: GPS

Genome Polysemy and Synonymy (GPS; unpublished) is a visualization platform for the analysis of alignments between optical maps, optical contigs, and *in silico* restriction maps created from sequence data. The software takes an xml file consisting of several optical maps and their alignments, and converts them into an interactive graphical representation using Scalable Vector Graphics (SVG). The SVG engine within GPS enables users to zoom in/out, pan, arbitrarily position optical maps, or contigs, and highlight selected features in ways designed to greatly enhance visual analysis of alignments. Such advantages allow users to more fully understand compound events involving translocations, inversions, and frank aberrations, or discordances. GPS visualization capabilities are based on an Open Source SVG manipulating library called Apache Batik (http://xmlgraphics.apache.org/batik/), and the last version of Java (1.8). One of the most useful advantages of the software is its ability to efficiently process and render very large map alignments within sizable and complex genomes (~3 Gb). GPS source code is accessible here: https://github.com/schwartz-lab/genome-polysemy-and-synonymy

### DNA sequencing

One lane of 150 bp PE Illumina sequencing was performed from blood extracted genomic DNA from Dominette L1 014490 to generate 515 million reads (the SRA archive number in NCBI: SRP05124). Reads were mapped to assembly UMD3.1 using CLC Bio Genomics Workbench software (CLC Bio, Aarhus, Denmark; 85 % of the reads mapped to UMD3.1) using the following settings: mismatch cost = 2; linear gap cost for insertions and deletions = 3; length fraction = 0.6; similarity fraction = 0.9; auto detect pair distance and ignore non-specific matches.

### Ethics statement

The bovine blood sample used is the property of the ARS USDA, therefore, no specific permits were required for the described studies.

## Additional files

Additional file 1: Figure S1. Rmap alignments (“hits”) against UMD3.1 for each chromosome; colored hash marks represent aligned Rmaps and annotated by tallies of coverage (X) and total mass (Mb). Rmap alignment for each chromosome is shown at the end of each chromosome. Green box (21,500,000–24,800,000 bp) highlights a 3.3 Mb region harboring dense Rmap alignments. Purple boxes (chr7:7,800,000–22,500,000 bp; chr12:70,360,000–76,785,000 bp) show regions of diminished Rmap alignments, suggesting that the sequence assemblies here are likely problematic. (PDF 16691 kb) Additional file 2: Figure S2. Rmap alignments (“hits”) against Btau4.6 for each chromosome; colored hash marks represent aligned Rmaps and annotated by tallies of coverage (X) and total mass (Mb). Green box (21,500,000–24,800,000 bp) highlights a 3.3 Mb region with sparse Rmap alignments. (PDF 10574 kb) Additional file 3: Table S1. Tabulation of discordances between BtOM1.0 and UMD3.1. (XLSX 399 kb) Additional file 4: Table S2. Tabulation of discordances between BtOM1.0 and Btau4.6. (XLSX 625 kb) Additional file 5: Figure S3. Circos plot of the discordances between BtOM1.0 optical maps and the *in silico* maps of the Btau4.6 sequence assembly. The tracks A, B, C, D, E, and F represents the six categories of discordances: Insertions (INSs), deletions (DELs), complex multiple events including insertion/deletion/inversion etc.(COMPLEXs), missing restriction sites (MCs), extra restriction sites (ECs), and large inversion or translocations (ITs). (EPS 3642 kb)

## Abbreviations

BAC: Bacterial Artificial Chromosome
BtOM1.0: *Bos taurus* optical map version 1.0
Btau4.6: *Bos taurus* genome sequence build 4.6 from Baylor College of Medicine Human Genome Sequence Center
COMPLEX: Sequence misassembly based on optical map and sequence comparison
DEL: Extraneous sequences based on optical map and sequence comparison
EC: Extra restriction cuts based on optical map and sequence comparison
EDTA: Ethylenediaminetetraacetic acid
FPC: FingerPrint Contig
G & G: "Germinate and Grow” for *de novo* assembly algorithm
GPS: Genome Polysemy and Synonymy software
HMW: High Molecular Weight
INS: Missing sequences based on optical map and sequence comparison
IT: Inverted/Translocated sequences based on optical map and sequence comparison
MC: Missing restriction cuts based on optical map and sequence comparison
UMD3.1: Bovine genome sequence build 3.1 from the Center for Bioinformatics and Computational Biology at the University of Maryland
Rmap: Raw single molecule optical Maps
SRA: Sequence Read Archive
SVG: Scalable Vector Graphics
WGS: Whole Genome Shotgun
