# Supplementary material for: A clone-free, single molecule map of the domestic cow (Bos taurus) genome
Source: BMC Genomics. 2015 Aug 28;16(1):644. doi: 10.1186/s12864-015-1823-7 (PMC4551733; doi:10.1186/s12864-015-1823-7)
Supplement: Additional file 1: Figure S1. — Rmap alignments (“hits”) against UMD3.1 for each chromosome; colored hash marks represent aligned Rmaps and annotated by tallies of coverage (X) and total mass (Mb). Rmap alignment for each chromosome is shown at the end of each chromosome. Green box (21,500,000–24,800,000 bp) highlights a 3.3 Mb region harboring dense Rmap alignments. Purple boxes (chr7:7,800,000–22,500,000 bp; chr12:70,360,000–76,785,000 bp) show regions of diminished Rmap alignments, suggesting that the sequence assemblies here are likely problematic. (PDF 16691 kb) [file 12864_2015_1823_MOESM1_ESM.pdf]

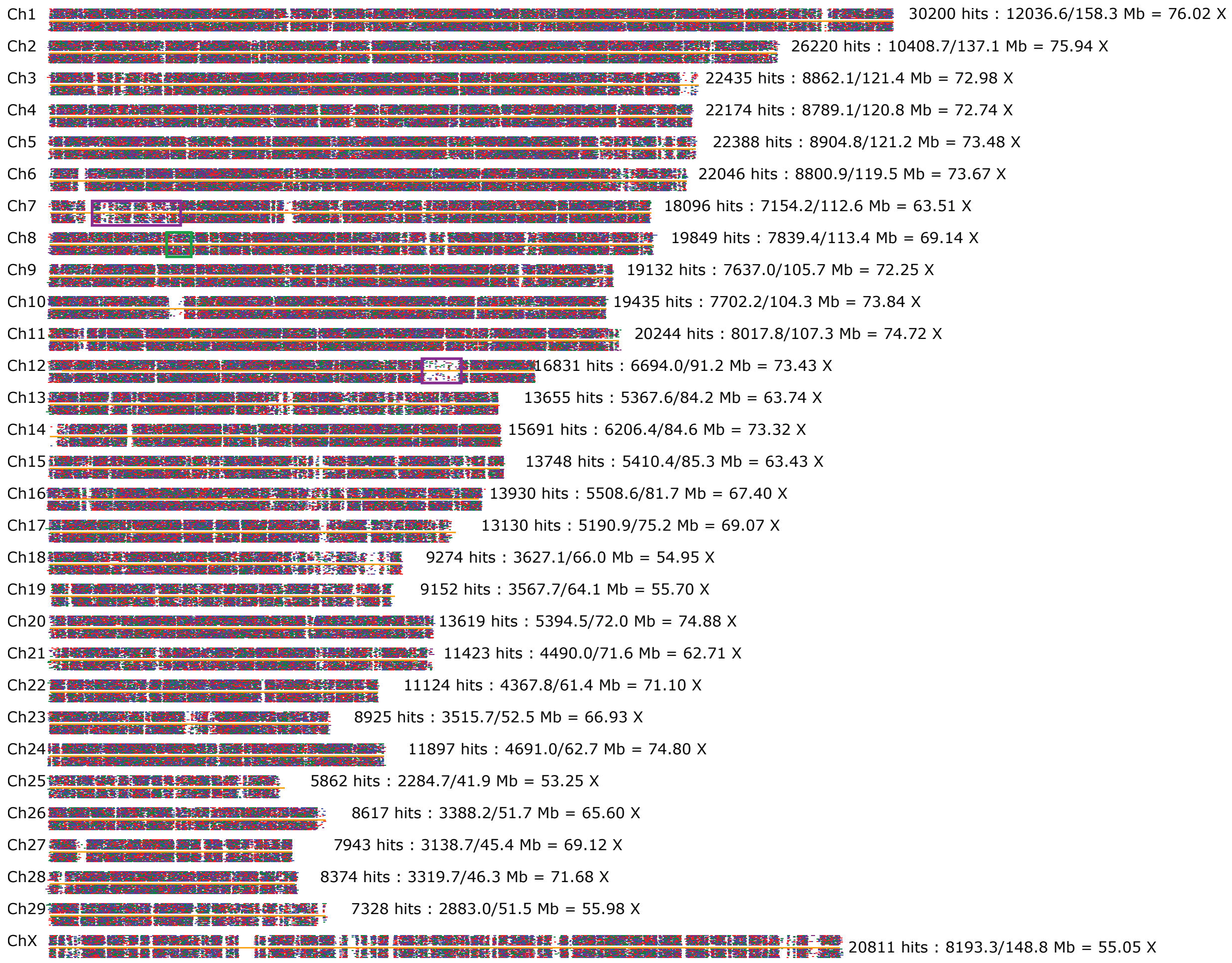

Ch1 30200 hits : 12036.6/158.3 Mb = 76.02 X
Ch2 26220 hits : 10408.7/137.1 Mb = 75.94 X
Ch3 22435 hits : 8862.1/121.4 Mb = 72.98 X
Ch4 22174 hits : 8789.1/120.8 Mb = 72.74 X
Ch5 22388 hits : 8904.8/121.2 Mb = 73.48 X
Ch6 22046 hits : 8800.9/119.5 Mb = 73.67 X
Ch7 18096 hits : 7154.2/112.6 Mb = 63.51 X
Ch8 19849 hits : 7839.4/113.4 Mb = 69.14 X
Ch9 19132 hits : 7637.0/105.7 Mb = 72.25 X
Ch10 19435 hits : 7702.2/104.3 Mb = 73.84 X
Ch11 20244 hits : 8017.8/107.3 Mb = 74.72 X
Ch12 16831 hits : 6694.0/91.2 Mb = 73.43 X
Ch13 13655 hits : 5367.6/84.2 Mb = 63.74 X
Ch14 15691 hits : 6206.4/84.6 Mb = 73.32 X
Ch15 13748 hits : 5410.4/85.3 Mb = 63.43 X
Ch16 13930 hits : 5508.6/81.7 Mb = 67.40 X
Ch17 13130 hits : 5190.9/75.2 Mb = 69.07 X
Ch18 9274 hits : 3627.1/66.0 Mb = 54.95 X
Ch19 9152 hits : 3567.7/64.1 Mb = 55.70 X
Ch20 13619 hits : 5394.5/72.0 Mb = 74.88 X
Ch21 11423 hits : 4490.0/71.6 Mb = 62.71 X
Ch22 11124 hits : 4367.8/61.4 Mb = 71.10 X
Ch23 8925 hits : 3515.7/52.5 Mb = 66.93 X
Ch24 11897 hits : 4691.0/62.7 Mb = 74.80 X
Ch25 5862 hits : 2284.7/41.9 Mb = 53.25 X
Ch26 8617 hits : 3388.2/51.7 Mb = 65.60 X
Ch27 7943 hits : 3138.7/45.4 Mb = 69.12 X
Ch28 8374 hits : 3319.7/46.3 Mb = 71.68 X
Ch29 7328 hits : 2883.0/51.5 Mb = 55.98 X
ChX 20811 hits : 8193.3/148.8 Mb = 55.05 X
