# Supplementary material for: A clone-free, single molecule map of the domestic cow (Bos taurus) genome
Source: BMC Genomics. 2015 Aug 28;16(1):644. doi: 10.1186/s12864-015-1823-7 (PMC4551733; doi:10.1186/s12864-015-1823-7)
Supplement: Additional file 2: Figure S2. — Rmap alignments (“hits”) against Btau4.6 for each chromosome; colored hash marks represent aligned Rmaps and annotated by tallies of coverage (X) and total mass (Mb). Green box (21,500,000–24,800,000 bp) highlights a 3.3 Mb region with sparse Rmap alignments. (PDF 10574 kb) [file 12864_2015_1823_MOESM2_ESM.pdf]

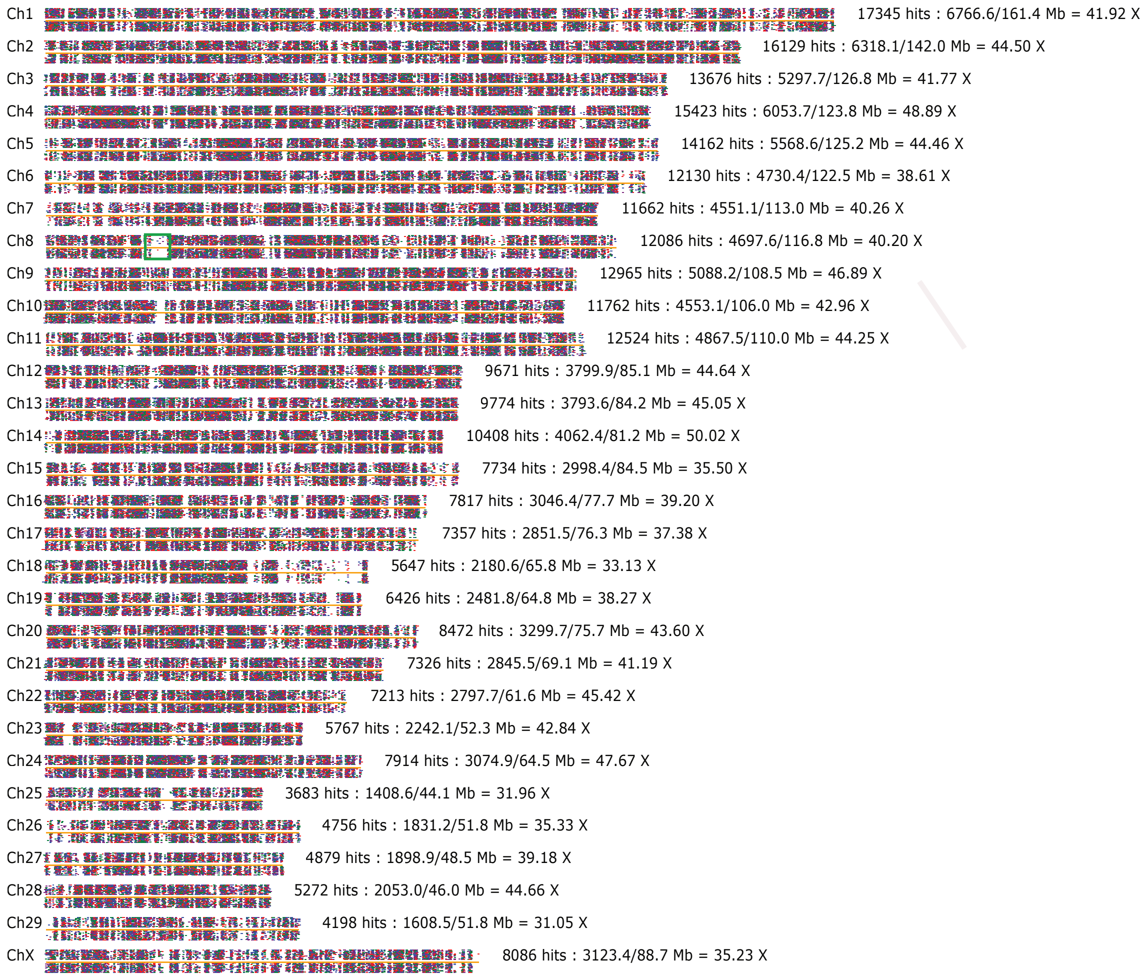

Ch1 17345 hits : 6766.6/161.4 Mb = 41.92 X
Ch2 16129 hits : 6318.1/142.0 Mb = 44.50 X
Ch3 13676 hits : 5297.7/126.8 Mb = 41.77 X
Ch4 15423 hits : 6053.7/123.8 Mb = 48.89 X
Ch5 14162 hits : 5568.6/125.2 Mb = 44.46 X
Ch6 12130 hits : 4730.4/122.5 Mb = 38.61 X
Ch7 11662 hits : 4551.1/113.0 Mb = 40.26 X
Ch8 12086 hits : 4697.6/116.8 Mb = 40.20 X
Ch9 12965 hits : 5088.2/108.5 Mb = 46.89 X
Ch10 11762 hits : 4553.1/106.0 Mb = 42.96 X
Ch11 12524 hits : 4867.5/110.0 Mb = 44.25 X
Ch12 9671 hits : 3799.9/85.1 Mb = 44.64 X
Ch13 9774 hits : 3793.6/84.2 Mb = 45.05 X
Ch14 10408 hits : 4062.4/81.2 Mb = 50.02 X
Ch15 7734 hits : 2998.4/84.5 Mb = 35.50 X
Ch16 7817 hits : 3046.4/77.7 Mb = 39.20 X
Ch17 7357 hits : 2851.5/76.3 Mb = 37.38 X
Ch18 5647 hits : 2180.6/65.8 Mb = 33.13 X
Ch19 6426 hits : 2481.8/64.8 Mb = 38.27 X
Ch20 8472 hits : 3299.7/75.7 Mb = 43.60 X
Ch21 7326 hits : 2845.5/69.1 Mb = 41.19 X
Ch22 7213 hits : 2797.7/61.6 Mb = 45.42 X
Ch23 5767 hits : 2242.1/52.3 Mb = 42.84 X
Ch24 7914 hits : 3074.9/64.5 Mb = 47.67 X
Ch25 3683 hits : 1408.6/44.1 Mb = 31.96 X
Ch26 4756 hits : 1831.2/51.8 Mb = 35.33 X
Ch27 4879 hits : 1898.9/48.5 Mb = 39.18 X
Ch28 5272 hits : 2053.0/46.0 Mb = 44.66 X
Ch29 4198 hits : 1608.5/51.8 Mb = 31.05 X
ChX 8086 hits : 3123.4/88.7 Mb = 35.23 X
